# JAK Inhibitors: A Double‐Edged Sword in Immune‐Mediated Diseases Management

**DOI:** 10.1002/ueg2.12716

**Published:** 2024-11-29

**Authors:** Luisa Bertin, Edoardo Vincenzo Savarino

**Affiliations:** ^1^ Gastroenterology Unit Azienda Ospedale Università Padova Padova Italy; ^2^ Department of Surgery, Oncology and Gastroenterology University of Padova Padova Italy; ^3^ Humanitas Clinical and Research Center IRCCS Rozzano Italy

**Keywords:** gastrointestinal perforation, JAK inhibitors, rheumatoid arthritis, safety

## Abstract

JAK inhibitors are pivotal in treating immune‐mediated inflammatory diseases (IMIDs) like rheumatoid arthritis (RA) and inflammatory bowel disease. However, emerging safety concerns warrant careful evaluation. A recent analysis of the FDA Adverse Event Reporting System (FAERS) revealed that RA patients using JAK inhibitors face nearly double the risk of gastrointestinal perforations (GIPs) compared to those on biologics, particularly with concurrent steroid or NSAID use. Additionally, the FDA's ORAL Surveillance study linked tofacitinib with higher rates of cancer and cardiovascular events, prompting regulatory restrictions. These findings highlight the importance of balancing JAK inhibitors' therapeutic benefits with potential risks, emphasizing the need for patient‐centred risk assessment and vigilant monitoring to optimize outcomes.

Over the past decade, there has been increasing interest in clinical immunology and rheumatology in developing targeted therapies to inhibit cytokines and their signalling pathways. This has led to the creation and adoption of Janus kinase (JAK) inhibitors [1]. These inhibitors, which target cytokine‐activated transmembrane receptors such as JAK1, JAK2, JAK3, and TYK2, act via the JAK‐STAT pathway, a critical regulator of immune function, cell proliferation, and haematopoiesis.

JAK inhibitors have significantly transformed the treatment landscape for immune‐mediated inflammatory diseases (IMIDs) such as rheumatoid arthritis (RA), psoriatic arthritis, juvenile idiopathic arthritis, axial spondylarthritis, inflammatory bowel disease, vitiligo, atopic dermatitis, and alopecia areata [2, 3]. These agents have also shown promise in other conditions, including myelofibrosis, glucocorticoid‐refractory acute graft‐versus‐host disease, polycythaemia vera, and refractory peripheral T‐cell lymphoma [4]. However, recent research underscores the need for caution, given potential risks such as gastrointestinal perforations (GIPs), cancer and cardiovascular events linked to the use of some JAK inhibitors.

In this issue of the *United European Gastroenterology Journal* an interesting article examined data from the Food and Drug Administration (FDA)'s Adverse Event Reporting System (FAERS), revealing that RA patients on JAK inhibitors had nearly double the risk of experiencing GIPs compared to those using biologic therapies. The study included 399,983 RA patients, out of whom 76,446 were treated with JAK inhibitors and 323,537 with biologic DMARDs (bDMARDs), including anti–tumour necrosis factor alpha (anti‐TNF‐*α*) therapy, rituximab, and abatacept. Compared to bDMARDs, JAK inhibitors showed a significantly increased reporting of GIPs, with an adjusted reporting odds ratio (adj. ROR) of 1.98 [1.69–2.31]. Moreover, patients with concurrent steroid or NSAID use had an even higher risk (adj. ROR = 2.82 [2.41–3.31]).

In addition to safety concerns, the FDA ORAL Surveillance study associated tofacitinib, a commonly prescribed JAK inhibitor, with increased risks of cancer and cardiovascular events [5]. This randomized, open‐label study included 4362 patients aged 50 and older, all of whom had at least one cardiovascular risk factor. Researchers compared the safety profile of tofacitinib against anti‐TNF‐*α* therapy, finding significantly higher rates of malignancies and major adverse cardiovascular events (MACE) among patients taking tofacitinib. Specifically, malignancy rates were 5.8 per 100 patient‐years for tofacitinib versus 3.9 for anti‐TNF‐*α* therapy, while MACE rates were 4.4 per 100 patient‐years compared to 2.0. Consequently, the FDA and European Medicines Agency (EMA) have restricted the use of JAK inhibitors for patients without suitable alternative treatment options, particularly in those aged 65 and older or with elevated risks of MACE (such as myocardial infarction and stroke) and cancer, including those with a history of smoking or prolonged smoking exposure.

Three key considerations arise from these findings. First, it is crucial to contextualize the risks of JAK inhibitors within the broader treatment spectrum for IMIDS, particularly for RA. Every treatment option, from steroids to anti–TNF‐*α* therapies and thiopurines, carries their own significant risks. Long‐term steroid use, for instance, is associated with severe side effects like osteoporosis, diabetes, and hypertension, while combining anti–TNF‐*α* therapies with thiopurines has been linked to an increased risk of haematological malignancies such as lymphoma [6, 7]. Thus, treatment decisions should consider the comprehensive risk‐benefit profiles of all available options.

Second, it is essential to understand the difference between absolute and relative risk. The study's findings indicate an adj. ROR of approximately 2.0 for GIPs among JAK inhibitor users compared to those on biologics. While this relative increase suggests caution, it does not translate to a high absolute risk for each patient. The reported 230 cases of GIPs among 76,446 JAK inhibitor users indicate an absolute risk of approximately 0.3%, highlighting the importance of distinguishing between absolute and relative risks when evaluating safety data.

Third, the potential adverse events associated with JAK inhibitors must be weighed against the impact of chronic disease on disability and quality of life. IMIDs have a profound effect on physical, emotional, and social well‐being [8]. Therefore, shared decision‐making between patients and healthcare providers is essential in this context.

Pharmacovigilance studies that leverage FAERS data offer valuable insights into drug safety in real‐world settings, often identifying risks that may not be fully apparent in controlled clinical trials. However, it is essential to recognize that studies based on large databases are prone to underreporting and other biases, including those from non‐healthcare personnel. Furthermore, it is not possible to fully rule out the influence of unaccounted confounding factors. Nevertheless, as the use of JAK inhibitors expands, both healthcare providers and patients must remain vigilant regarding potential adverse effects. While the findings do not advocate for abandoning JAK inhibitors, they emphasize the need for careful patient selection, optimized dosing, and regular monitoring. For some patients, especially those requiring long‐term steroid or NSAID therapy, biologic therapies may present a safer alternative.

In conclusion, JAK inhibitors represent a significant advancement in treating autoimmune and inflammatory diseases. A balanced approach that carefully weighs their benefits against potential risks is essential. By fostering open communication, selecting patients judiciously, and monitoring for adverse events, healthcare providers can optimize the therapeutic potential of JAK inhibitors while minimizing associated risks.

## Author Contributions

L.B. and E.V.S.: collection and analysis of the data, draft of the manuscript, approving final version.

## Conflicts of Interest

L.B. has nothing to declare. E.V.S. has served as speaker for Abbvie, Abivax, Agave, AGPharma, Alfasigma, Apoteca, Biosline, CaDiGroup, Celltrion, Dr Falk, EG Stada Group, Fenix Pharma, Galapagos, Johnson&Johnson, JB Pharmaceuticals, Innovamedica/Adacyte, Eli Lilly, Malesci, Mayoly Biohealth, Montefarco, Novartis, Omega Pharma, Pfizer, Rafa, Reckitt Benckiser, Sandoz, Sanofi/Regeneron, SILA, Sofar, Takeda, Tillots, Unifarco; E.V.S. has served as consultant for Abbvie, Agave, Alfasigma, Biogen, Bristol‐Myers Squibb, Celltrion, Dr. Falk, Eli Lilly, Fenix Pharma, Ferring, Giuliani, Grunenthal, Johnson&Johnson, JB Pharmaceuticals, Merck & Co, Nestlè, Pfizer, Reckitt Benckiser, Sanofi/Regeneron, SILA, Sofar, Takeda, Unifarco; EVS received research support from Bonollo, Difass, Pfizer, Reckitt Benckiser, Sanofi/Regeneron, SILA, Sofar, Unifarco, Zeta Farmaceutici. Brigida Barberio has served as a speaker for Abbvie, Agave, Alfasigma, AGpharma, Janssen, Lilly, MSD, Pfizer, Sofar, Takeda, and Unifarco. The other authors declare no conflict of interest.

## Data Availability

Data sharing not applicable to this article as no datasets were generated or analyzed during the current study.
